# Managing and controlling diseases transmitted by *Aedes* mosquitoes: a review on best practices

**DOI:** 10.1186/s41182-025-00890-7

**Published:** 2026-01-09

**Authors:** Rasoul Ebrahimi, Seyed Aria Nejadghaderi, Mohammad Khalili, AliAkbar Haghdoost, Abbas Aghaei-Afshar, Hamid Sharifi

**Affiliations:** 1 School of Medicine, Shahid Beheshti University of Medical Sciences, Tehran, Iran; 2 HIV/STI Surveillance Research Center, and WHO Collaborating Center for HIV Surveillance, Institute for Futures Studies in Health, Kerman University of Medical Sciences, Kerman, Iran; 3 Knowledge Hub for Migrant and Refugee Health, Institute for Futures Studies in Health, Kerman University of Medical Sciences, Kerman, Iran; 4 Department of Pathobiology, Faculty of Veterinary Medicine, Shahid Bahonar University of Kerman, Kerman, Iran; 5 Modeling in Health Research Center, Institute for Futures Studies in Health, Kerman University of Medical Sciences, Kerman, Iran; 6 Research Center of Tropical and Infectious Diseases, Kerman University of Medical Sciences, Kerman, Iran; 7 Institute for Global Health Sciences, University of California, San Francisco, CA USA

**Keywords:** Dengue fever, Best practice, *Aedes* control, World Health Organization, Integrated vector management, Public health, Review

## Abstract

**Background:**

Dengue fever (DF) is a viral disease caused by the dengue virus and is transmitted to humans by *Aedes* mosquitoes. It is characterized by symptoms such as high fever and severe headache, which can lead to severe complications. As there are no effective treatments or vaccines, prevention is crucial. This study aims to identify best practices from various countries for controlling *Aedes* populations and managing DF.

**Methods:**

We reviewed best practices for DF and *Aedes* mosquito control from various countries, including Taiwan, India, Oman, Singapore, Malaysia, Sri Lanka, Indonesia, Pakistan, China, the Philippines, Japan, Brazil, Paraguay, France, Portugal, Spain, Peru, the United States, Colombia, Australia, and Iran. The PubMed database was searched until August 2024.

**Results:**

Dengue outbreaks necessitate diverse control strategies across nations. Machine learning models incorporating climatic and entomological data improved outbreak prediction accuracy by up to 29%, while *Wolbachia*-based interventions reduced dengue incidence by 77% in urban trials. Community-driven programs enhanced preventive behaviors by 50–70%, and novel vaccines demonstrated > 94% efficacy against severe dengue. Challenges such as insecticide resistance and climate variability underscore the need for adaptive surveillance and cross-sector collaboration. Innovations in mobile health tools and sterile insect techniques further optimized vector control, achieving > 90% reductions in mosquito populations, compared with baseline densities before intervention.

**Conclusions:**

Effective *Aedes* mosquito management against DF requires community engagement, surveillance, and innovative control methods. Successful strategies from selected countries highlight the importance of interventions, ongoing research, and public education to reduce disease risks. Continuous research, collaboration across sectors, and public awareness are necessary to reduce *Aedes* mosquitoes’ risks and protect public health from vector-borne diseases.

**Supplementary Information:**

The online version contains supplementary material available at 10.1186/s41182-025-00890-7.

## Introduction

*Aedes* mosquitoes are well-known carriers of several harmful arboviruses, such as dengue, Zika, yellow fever, and chikungunya. These diseases significantly affect human health, leading to high rates of morbidity and mortality in many parts of the world. The complex nature of diseases transmitted by *Aedes* mosquitoes requires detailed and focused control strategies to reduce their impact effectively [1]. *Aedes* mosquitoes have evolved features that enhance their ability to transmit viruses, such as a preference for humans, an affinity for small containers as breeding sites, feeding during the day, and various strategies for seeking hosts. Urbanization and climate change worsen the situation by increasing the availability of suitable breeding environments and favorable conditions for these vectors to flourish [2]. Their function as vectors is related to their capacity to support viral growth, become infected, and subsequently transmit the virus to a susceptible host. This is referred to as vectorial competence. Additionally, it involves the virus’s ability to overcome the mosquito’s immune defenses [3].

The intricate dynamics of arboviruses vectored by *Aedes* mosquitoes require well-rounded and specific control measures to mitigate their effects effectively. Dengue fever (DF), in particular, stands out as an escalating threat among arboviral diseases and is mostly found in tropical and subtropical areas, with a risk of spreading to new regions. Over the past 40 years, it has significantly impacted global health and economies [4], affecting approximately 390 million people annually. Of these, around 96 million had symptoms, leading to 500,000 hospitalizations and 25,000 deaths each year [5]. The incidence of DF has risen since 1980, particularly in Asia, South America, and the Caribbean, due to four distinct serotypes. During the nineteenth century, DF epidemics were observed in various parts of Africa, beginning with the Zanzibar Islands in 1823 and again in 1870, followed by Burkina Faso in 1925 and South Africa between 1926 and 1927 [6]. By the 1960s, many laboratory-confirmed DF cases had been reported across continents [6]. There are four unique serotypes of the virus that can lead to a spectrum of symptoms, from mild fever to more severe manifestations, such as dengue hemorrhagic fever and dengue shock syndrome. Although the first infection with one serotype provides lifelong immunity, subsequent infections with different serotypes or more aggressive strains increases the likelihood of developing severe illness. This highlights the nature of dengue infections and their possible repercussions [7]. Transmission primarily occurs through the *Aedes aegypti* (*Ae. aegypti*) mosquito, but the virus can also spread via other *Aedes* species, such as *Aedes albopictus* (*Ae. albopictus*). Efforts are ongoing to control and prevent outbreaks globally [8].

Currently, there are no effective vaccines or antiviral therapies for DF, with Dengvaxia being the sole vaccine approved in the United States. However, the World Health Organization (WHO) advises against its use in seronegative individuals despite its approval in 20 other countries [9]. Owing to the lack of effective treatments, controlling mosquito populations is the main strategy for preventing dengue [10]. To combat vector-borne diseases, WHO launched the Global Vector Control Response for 2017–2030 [11], which is a framework for member nations to evaluate and formulate national policies for vector control requirements. Given the ever-changing vector distribution patterns, a continuous assessment of these strategies is essential [12]. Moreover, recent research suggests that specific microorganisms such as Wolbachia can greatly diminish dengue transmission. A three-year study conducted in Indonesia revealed a 76% decrease in dengue cases when *Ae. aegypti* mosquitoes were infected with Wolbachia [13].

Despite the increasing global burden of *Aedes* mosquito-borne diseases, there remains a notable gap in the literature regarding the effectiveness of various management and control interventions across different geographical and socioeconomic contexts, particularly in the 21 selected countries that will be the focus of this review. While numerous localized studies have explored specific control measures, comprehensive reviews that compare and consolidate these findings across countries remain limited. This review presents country-specific strategies for managing and controlling *Aedes* mosquitoes and the arboviruses they vector, drawing on experiences from 21 countries, including Taiwan, India, Oman, Singapore, Malaysia, Sri Lanka, Indonesia, Pakistan, China, the Philippines, Japan, Brazil, Paraguay, France, Portugal, Spain, Peru, the United States, Colombia, Australia, and Iran. Rather than providing a unified synthesis or ranking of interventions, the review compiles successful practices and innovative approaches as applied in varied geographical and socioeconomic settings. These examples are intended to support policymakers, public health officials, and researchers by illustrating practical methods that have been implemented globally, thereby contributing to ongoing efforts in vector control and public health improvement.

## Methods

This study focused on effective strategies to prevent and manage DF and *Aedes* mosquitoes across various countries, including Taiwan, India, Oman, Singapore, Malaysia, Sri Lanka, Indonesia, Pakistan, China, Philippines, Japan, Brazil, Paraguay, France, Portugal, Spain, Peru, the United States, Colombia, Australia, and Iran. Studies were selected based on their methodological rigor, clarity in describing intervention outcomes, and relevance to dengue or *Aedes* control. Articles were considered a best practice if they reported measurable improvements in either entomological (e.g., larval indices, adult mosquito density) or epidemiological indicators (e.g., dengue incidence, hospitalization rate) compared with baseline or control areas. We prioritized studies employing experimental, quasi-experimental, or community-based designs that demonstrated effectiveness in real-world settings. Countries were selected based on four main criteria: (1) high dengue burden or history of recent outbreaks with major public health impact; (2) geographic diversity across continents; (3) availability of published data describing intervention outcomes; and (4) inclusion of innovative or emerging approaches identified through expert input. We conducted searches on PubMed until August 2024 for published articles to identify best practices and included studies that addressed various interventions used to manage and control *Aedes* mosquitoes (Table S1). Our focus was on their effectiveness in managing *Aedes* mosquitoes and the diseases they transmit, including DF, from identified sources in the 21 selected countries. To improve transparency and to ensure appropriate representation for very large and heterogeneous countries, the search strategies and inclusion criteria were re-evaluated after an initial screening phase. We prioritized studies that described national programmes, multicenter trials, scalable interventions, or well-designed regional studies with clear epidemiological or operational outcomes, and we avoided listing every local practice to focus on transferable best practices. For countries with substantial internal heterogeneity (for example, India and Brazil), we included exemplar regional studies that illustrate scalable approaches (community-based environmental management, municipal integrated vector management, Wolbachia pilot deployments, or large multicenter trials). Furthermore, while the scope of this review was global, the country selection was limited to those with publicly available, peer-reviewed documentation of *Aedes* control strategies and outcomes. Several African nations are actively implementing vector management programs; however, consistent and detailed data from these settings were scarce in the indexed literature at the time of review. Therefore, countries were included primarily based on data completeness and the availability of verifiable “best practice” reports. The underrepresentation of African countries reflects a gap in published evidence rather than an absence of vector control activities, underscoring the need for further research in the region. For clarity and comparability, we categorized each country according to the predominant epidemiological pattern of *Aedes*-borne disease observed in published surveillance reports and peer-reviewed studies. Categories were assigned using operational criteria based on multi-year surveillance data, reports of autochthonous transmission, and documented outbreak history. Countries in the Results are presented grouped by epidemiological category, with countries listed alphabetically within each group. For each country, we reported the major control interventions described in the included studies.

## Results

### Overview of global strategies for *Aedes* mosquito and disease management

Effective management of *Aedes* mosquitoes and diseases they transmit relies on a layered, globally informed approach merging scientific innovation, localized action, and policy agility. Central to these efforts is vector control, which prioritizes environmental management, eliminating breeding sites through improved sanitation, drainage, and larvicidal treatments, and leverages novel tools such as Wolbachia-infected mosquitoes to suppress populations or block viral transmission, alongside Sterile Insect Techniques (SIT) and auto-dissemination devices for targeted insecticide delivery. Surveillance and predictive modeling further enhance precision, with machine learning, geographic information systems, and climate data enabling real-time outbreak forecasting and risk mapping, while mobile platforms streamline data collection and community reporting. Community-driven strategies bridge policy and practice. Health education campaigns can improve public awareness about dengue symptoms and prevention while addressing socioeconomic barriers. Local leaders and schools often spearhead these initiatives, fostering ownership and sustainability. Vaccination and clinical advances complement these measures. Advances in vaccination, exemplified by the tetravalent TAK-003 vaccine with > 90% efficacy against severe dengue, and improved clinical management underscore progress in reducing mortality, though universal vaccine coverage remains a challenge. These approaches are unified by cross-sector collaboration, where governments, healthcare systems, and international bodies align policies on integrated vector management, a framework coordinating health, environmental, and urban planning sectors to address outbreak root causes, and "One Health" principles, which recognize the interdependence of human, animal, and ecosystem health. Legal mandates reinforce compliance with sanitation standards and travel-related quarantine protocols, while insecticide resistance monitoring programs enable adaptive chemical control strategies. Climate resilience planning further ensures systems withstand environmental shifts that amplify disease risks. These strategies thrive on adaptability. For instance, Wolbachia releases may prioritize urban hotspots in one country, while another region might focus on SIT in rural areas. The following sections explore country-specific implementations, with a focus on a selection of methods and policies that have been implemented in the selected countries. Table 1 summarizes the key country-specific insights and best practices for *Aedes* mosquito and dengue control identified in this review, highlighting diverse approaches, innovations, and outcomes across different regions.

**Table 1:** Summary of key insights and best practices for *Aedes* mosquito and dengue control by country

Country	Key insights/best practices
Taiwan	Advanced predictive systems using machine learning and random forest models improved outbreak forecasting accuracy. Quarantine stations at airports effectively identified imported cases. Active and sticky traps showed higher sensitivity for early detection
India	Strengthening reporting systems and diagnostic capacity remains essential. Community-based education programs significantly improved awareness and preventive behaviors. Multicenter field trials with attracticide C21 reduced larval indices effectively
Oman	First local dengue transmissions highlighted weak surveillance and control measures. geographic information systems and Wolbachia-based approaches were recommended to enhance vector control
Singapore	A long-standing national dengue program emphasizes source reduction and environmental management. Large-scale releases of Wolbachia-infected males reduced dengue incidence by up to 77%. Predictive risk models and vaccine research (TAK-003) further strengthened control efforts
Malaysia	Field populations of *Aedes albopictus* from 14 regions were susceptible to all tested insect growth regulators, indicating their potential as effective and environmentally safer alternatives to conventional insecticides
Sri Lanka	Implementation of WHO-aligned strategies reduced mortality to 0.2%. Mobile-based Buzz-Mo system improved surveillance efficiency. Studies on sterile insect techniques (optimal dose 50 Gy) and Wolbachia presence supported sustainable vector control
Indonesia	Cluster-randomized trials in Yogyakarta showed an 83% reduction in dengue incidence following Wolbachia releases. Nationwide modeling predicted prevention of over 80% of dengue-related deaths. Community-driven ecosystem programs enhanced local ownership
Pakistan	Integrated vector management through improved sanitation, digital tracking, and household surveillance led to a 70% drop in dengue cases. Cross-sector coordination and community training were key to success
China	Community-based interventions in Guangzhou reduced dengue cases by 47%. Use of unmanned aerial vehicle mapping improved breeding-site identification. A detection-response system achieved 99.8% specificity in outbreak identification
Philippines	Ae. aegypti breeds mainly in households and Ae. albopictus in cemeteries. Dengue incidence rises after rainfall peaks. Ovitraps proved highly sensitive for early detection of Aedes presence
Japan	Imported cases triggered local outbreaks (e.g., 2014). Rapid responses such as park closures effectively curtailed spread. Improved multilingual communication and outbreak readiness were emphasized for large events
Brazil	Resistance to temephos prompted adoption of integrated methods combining Wolbachia, larvicidal traps, and sterile insect techniques, resulting in up to 38% reduction in arboviral diseases. Long-term economic analyses projected major cost savings
Paraguay	The 2024 epidemic surge prompted creation of the TopaDengue project, which used community inspections and a phone-based reporting system for rapid case follow-up, improving outbreak response
France	Autochthonous cases in Réunion and northern France led to intensified door-to-door surveillance. Ultralow-volume and fogging methods varied in effectiveness, stressing the need for real-time vector monitoring
Portugal	The 2012 Madeira outbreak led to enhanced port disinfection and donor screening. Auto-dissemination of pyriproxyfen showed moderate success in larval control depending on local conditions
Spain	Ae. albopictus established since 2004. Integrated community programs combining larvicides and education reduced breeding sites. Pilot Wolbachia and sterile insect technique projects in Valencia demonstrated promising results
Peru	National responses combined larviciding, insecticide spraying, and triage units during outbreaks. Mobile technology improved household prevention. Spatial repellent trials achieved a 34% reduction in infections
United States	Rapid response in Key Largo (2020) used long-lasting larvicides and ultralow-volume spraying. Wolbachia male releases in Houston cut wild populations by ~ 93%. Biological controls like Utricularia macrorhiza showed strong larvicidal activity
Colombia	The SIVIEN AEDES platform enabled geospatial tracking of vector density and resistance. Wolbachia deployments reduced dengue incidence, while autocidal ovitraps (> 80% efficacy) and WhatsApp communication boosted community engagement
Australia	Pyrethroid spraying, copepod predators, and Rapid Surveillance for Vector Presence surveillance systems proved effective. Wolbachia releases in Queensland reduced dengue incidence by up to 95% within two years
Iran	Imported used tires pose a potential introduction route for Aedes species. Studies revealed knowledge gaps among healthcare workers, highlighting the need for continuous training and strengthened border surveillance

### East, South, and Southeast Asia

#### Taiwan

Control strategies in Taiwan focus on quarantine, vector management, and the predictive modeling of epidemics. Recent studies have utilized advanced technologies such as machine learning to improve outbreak forecasts. One study developed a dengue prediction model that integrated meteorological data, vector indices, and air quality assessments, and found that the random forest method performed best, achieving an area under the receiver operating characteristic curve of 0.95 for predicting dengue incidence. A temperature above 30 °C was identified as the key factor influencing dengue incidence, while air quality had a lesser but relevant correlation [14]. Another research effort focused on Kaohsiung using a new regression model to examine daily climate variability and its effect on dengue cases in 2014–2015. Humidity and mosquito activity were significant predictors, which provided real-time data for public health interventions [15]. Moreover, a two-stage risk prediction system combined high-resolution weather data and *Ae. aegypti* surveillance to evaluate dengue transmission risk. This approach increased accuracy by 29% and reduced prediction error by 6.9%, which recommended insights into geographical risk distribution [16].

A model designed to predict the time–space spread of a DF epidemic in Kaohsiung City in 2002 was effective in capturing user-defined parameters, particularly in urban areas near outbreak sources. However, its performance can be influenced by factors such as virus serotypes and human interventions that make it a valuable tool for government agencies [17]. Simultaneously, machine learning techniques were applied to identify emerging dengue hotspots in Taiwan, revealing three clusters associated with sea surface temperature anomalies, with an AlexNet-based convolutional neural network achieving 100% classification accuracy on the validation dataset for hotspot detection [18]. The Kaohsiung City Government responded to rising cases in 2016 by establishing quarantine referral stations at airports, that successfully identified positive cases among travelers, reduced local dengue instances, and emphasized the role of imported cases in local transmission [19]. A dynamic transmission model assessed infection risk in *Ae. aegypti* mosquitoes, confirming that mosquito biting rates are pivotal. The study indicated a 50% probability of increased dengue incidence when average temperatures ranged between 26 °C and 32 °C, suggesting that optimal environmental conditions for mosquito breeding coincide with higher disease transmission risk, thereby emphasizing the need for strengthened vector control and surveillance during such temperature ranges [20]. Furthermore, active traps were found to be more effective than passive traps in correlating mosquito capture rates with disease incidence [21], whereas sticky plastic traps demonstrated higher sensitivity in early dengue detection [22]. Active traps, such as fan-based or light-suction traps, actively draw mosquitoes using airflow or light to enhance capture efficiency, whereas passive traps rely on mosquitoes’ natural movement and landing behavior without mechanical attraction. Sticky plastic traps were shown to have higher sensitivity in early dengue detection because they continuously capture adult Aedes mosquitoes over time, allowing early identification of viral presence or vector density peaks before clinical case surges occur.

#### China

To effectively control *Aedes* mosquitoes and DF, collaborative commitments from governments, stakeholders, and communities are needed, emphasizing leadership and financial investment. Operational research is essential for evidence-based policymaking, exploring the factors that influence dengue transmission, and enhancing diagnostic techniques. Key strategies include increasing public awareness, developing integrated surveillance systems, and bridging the knowledge gaps [23]. Integrated vector management and understanding cultural and ecological contexts are crucial for effective implementation. The “One Health” approach should be implemented nationally, focusing on strategic land use and collaborations across sectors. Strengthening the water supply, drainage systems, and comprehensive health education is necessary, along with ongoing monitoring of *Aedes* sensitivity to insecticides. Collaborative policies in integrated dengue management are necessary for advancing global health security and achieving sustainable development goals, especially as the search for effective vaccines continues [24].

In 2014, Guangdong Province, China, experienced a significant DF outbreak, prompting an integrated community-based intervention in Guangzhou. This approach includes mosquito breeding eradication, adult mosquito control using insecticides, public health education, and community participation. Consequently, mosquito density decreased significantly, leading to a 47% reduction in reported dengue cases, and approximately 23,302 cases were prevented [25]. Further research in 2015 in the Xiangqiao area utilized a dynamic modeling approach to show that timely, stringent interventions could effectively minimize the outbreak’s size and duration, demonstrating the importance of early action in dengue control [26]. The effective control of *Aedes* mosquito populations involves identifying and eliminating potential breeding sites. A study focused on *Ae. albopictus* in the Yangtze River Basin developed a dataset of potential breeding sites, such as tanks, basins, and flowerpots. A spatial density model was used to identify regions with high breeding site density and areas requiring resource management. This research proposed utilizing unmanned aerial vehicles for mapping breeding sites, enhancing efficiency, and operational guidance for mitigation efforts [27]. Furthermore, a detection and response system based on historical data achieved 99.8% specificity in identifying dengue outbreaks in China, underscoring its utility in public health strategies [28]. Studies of *Aedes* mosquitoes infected with Wolbachia have also contributed to vector control knowledge [29]. Furthermore, an examination of the dengue spread during the COVID-19 pandemic in Yunnan Province revealed a significant reduction in cases, effective control of urban-to-suburban transmission, and limited circulation of multiple strains, highlighting the pandemic’s unintended benefit for dengue transmission dynamics [30].

#### Japan

Although Japan does not have domestic cases of DF, ongoing imports, primarily from South and Southeast Asia, combined with the presence of *Ae. aegypti* and *Ae. albopictus* mosquitoes, pose a risk for potential transmission [31]. Dengue was not seen until 2013, when a traveler brought the virus to Japan, leading to a notable outbreak in 2014 with 160 confirmed cases, signaling a renewed threat during the summer [32]. Government responses included aggressive mosquito control and public awareness campaigns, although these measures initially fell short, prompting the closure of Yoyogi Park to mitigate further transmission. Mathematical modeling demonstrated that while communication and mosquito control were essential, decisive actions such as park closures were particularly effective in curtailing the spread [33]. For the 2020 Tokyo Olympics and Paralympics, a study highlighted Japan’s robust infectious disease detection system, but identified gaps in training for medical professionals, multilingual communication, and emergency protocols for travelers, suggesting improvements for future outbreaks [34].

#### Philippines

A comprehensive study conducted between August 1985 and July 1987 in Manila examined the primary dengue vectors, *Ae. aegypti* and *Ae. albopictus*. The findings indicated that *Ae. aegypti* predominantly breeds in residential settings, whereas *Ae. albopictus* is more commonly found in cemeteries. The study evaluated the efficacy of various survey methods, revealing that ovitraps effectively detected *Ae. aegypti*, but were not suitable for tracking population fluctuations over time. Notably, dengue transmission was closely linked to rainfall patterns, with an increase in cases observed approximately two months after the rainy season began [35]. The research also highlighted the competitive limitations of *Ae. albopictus* in residential areas and noted similar peak biting activities for both species, peaking from 05:30–06:00 and 17:30–18:00 [36].

#### Indonesia

A study in Yogyakarta utilized a cluster-randomized design to assess the impact of releasing Wolbachia-infected *Ae. aegypti* mosquitoes on dengue transmission. Twenty-four clusters were split into the intervention and control groups, both maintaining routine mosquito control measures. The introduction of Wolbachia mosquitoes significantly decreased the incidence of symptomatic dengue and related hospitalizations [13]. A subsequent Cochrane review supported the efficacy of Wolbachia mosquitoes in endemic settings, although further studies are needed for generalizability and evaluation of acceptance and costs [37]. In addition, in Yogyakarta, dengue hemorrhagic fever cases decreased by 83% compared to untreated control areas in fully treated zones where Wolbachia releases were completed. In partially treated areas, DHF cases decreased by approximately 63.7% compared to untreated regions during the Wolbachia release period [38]. Modeling suggested that a nationwide Wolbachia release could prevent 86.2% of dengue-related deaths in the long term [39]. Moreover, a community-based intervention in Yogyakarta aimed at dengue ecosystem management has highlighted increased community awareness and participation. Six months post-intervention, significant improvements in knowledge, stakeholder collaboration, and community ownership in vector management were observed, indicating that community initiatives could offer more sustainable impacts than traditional top-down approaches [40].

#### Malaysia

Malaysia has a long-standing history of research and operational efforts in *Aedes* control, supported by strong national surveillance systems and active contributions from both academic institutions and the Ministry of Health [41]. Multiple studies have evaluated integrated vector management approaches, which highlight the country’s emphasis on environmental sanitation, community-driven source reduction, insecticide resistance monitoring, and biological control [41–43]. National entomological surveillance has documented widespread pyrethroid resistance in *Aedes aegypti* and *Aedes albopictus*, prompting the use of alternative insecticides and rotation strategies [44, 45]. Community-based COMBI (Communication for Behavioural Impact) programs, implemented in states such as Selangor, Penang, and Johor, have consistently improved household vector-control practices and reduced larval indices [46, 47].

Several Malaysian field studies have also assessed novel tools. For example, auto-dissemination of pyriproxyfen using ovitrap-based delivery systems has produced substantial reductions in *Aedes* populations in urban settings [48, 49]. Other work has explored biological agents, including predatory copepods and *Bacillus thuringiensis israelensis*, which have demonstrated strong efficacy and operational feasibility [50, 51]. In addition, Malaysia has maintained one of the region’s most comprehensive dengue early-warning systems, integrating climate data, case surveillance, and entomological indices to predict transmission hotspots [52–55]. These combined findings demonstrate a mature, multifaceted vector-control landscape that extends far beyond chemical interventions, positioning Malaysia as a regional leader in dengue control research and practice [41, 43, 56].

In recent years, Malaysia has begun implementing Wolbachia-based vector control, releasing *Aedes* mosquitoes infected with Wolbachia bacteria, which block virus replication and transmission. Field releases of *Aedes aegypti* carrying the wAlbB Wolbachia strain have led to substantial reductions in dengue incidence. One study across 20 release sites reported a 62.4% average drop compared to control sites, rising up to about 75.8% when Wolbachia frequency reached 100% [57]. The wAlbB strain is well-suited for Malaysia’s tropical climate and remains stable even at high temperatures [58]. Recent work in urban settings, such as high-rise buildings in Selangor, demonstrates the sustainability and feasibility of maintaining Wolbachia in *Aedes* populations under operational conditions [59]. The Wolbachia strategy represents a promising and increasingly implemented biological control tool complementing existing integrated vector-management efforts in Malaysia.

#### Singapore

The DF outbreak, which emerged in the 1960s, prompted the establishment of an extensive Dengue Control Program. This program successfully reduced dengue cases by tenfold in the 1990s and maintained lower levels, emphasizing source reduction and environmental management as primary strategies [60]. Collaboration among government ministries, communities, and the private sector enhances community engagement and ensures compliance through house-to-house inspections and legal frameworks [61]. Despite these successes, the program faces challenges owing to climate change and heightened population immunity. Innovations include Wolbachia-infected male mosquitoes and spatiotemporal risk assessment models. Furthermore, a two-stage framework for early warning systems has proven effective, using historical data to predict outbreaks and allow timely interventions, which has shown reliability in predicting dengue surges in 2013, 2014, and 2016 [62].

Due to the absence of effective treatments for DF, vector control is important. A study conducted in Singapore from 2018 to 2022 assessed the release of Wolbachia-infected male *Ae. aegypti* mosquitoes (wAlbB strain) to lower dengue incidence. Large-scale releases took place in the towns of Yishun and Tampines, alongside targeted interventions in Bukit Batok and Choa Chu Kang, utilizing the same dengue control protocols. The intervention showed up to 77% effectiveness, resulting in a reduction of 2242 dengue cases per 100,000 people in intervention sites, with consistent outcomes across demographics [63]. In addition, public acceptance was overwhelmingly positive, with residents expressing no concerns regarding modified mosquitoes [64]. Ongoing studies continue to explore the efficacy of this vector-control strategy [65]. Concurrent research focused on the tetravalent dengue vaccine, TAK-003, and found that higher neutralizing antibody titers were achieved with the high-potency formulation (TDV-HD) than with the lower potency version (TDV), with comparable safety profiles [66]. Furthermore, a phase 2 study on the booster effect of CYD-TDV showed that antibody titers increased post-vaccination but declined over 24 months alongside short-lived memory B cells [67, 68].

#### Sri Lanka

The nation’s response aligns with the WHO’s Global Strategy for Dengue Prevention and Control, which aims to reduce dengue mortality below 0.1% and morbidity by 50% by 2020 [69]. Key strategies are multisectoral collaboration, the establishment of a dedicated dengue control unit, and enhanced disease surveillance and clinical management. As a result, dengue mortality has decreased to 0.2% due to advanced real-time surveillance and improved diagnostic capabilities [70]. Research into the role of Wolbachia bacteria included a study in Jaffna, where *Ae. albopictus* mosquitoes were screened for Wolbachia infections. These findings revealed widespread infections with wAlbA and wAlbB strains, with genetic sequences showing variations from those in mainland Sri Lanka [71]. This suggests that Wolbachia’s presence may play an essential role in developing dengue control methods in the coastal areas of Sri Lanka.

In Sri Lanka, a study assessed the efficacy of the TAK-003 vaccine in participants aged 4–16 years, demonstrating an efficacy of 94.7% against virologically confirmed dengue cases and 95.7% against hospitalizations within 12 months following two doses. Modeling based on outbreak dynamics from 2017, with the assumption of 65% vaccination coverage and initiation 30 days prior, indicated that TAK-003 significantly reduced cases and flattened outbreak curves, emphasizing the importance of prompt campaign initiation and wide coverage [72]. Furthermore, the SIT is being tested for dengue control by releasing sterilized male *Ae. aegypti* mosquitoes to reduce populations. A study evaluating the effects of gamma radiation on mosquito cohorts revealed that while survival rates decreased with increasing radiation doses, they remained above 90% for controls, and flight ability was unaffected. An optimal radiation dose of 50 Gy was determined to produce sterile males for dengue suppression efforts in Sri Lanka [73]. Sri Lanka has underutilized mobile interventions in dengue management since the surge in mobile phone usage in 2009. To address public health workers’ needs in Colombo, an assessment was conducted through interviews and surveys with 29 workers to identify the challenges in current dengue surveillance practices. The findings indicated that existing paper-based methods were time-consuming, required 7–10 days for reporting, and faced issues such as delays in reporting mosquito breeding sites and low community participation. These insights led to the development of the Buzz-Mo mobile system, which enhances digital surveillance, disease mapping, and education and is now utilized by all 55 public health workers in Colombo [74].

#### India

A review emphasized effective control strategies for DF, recommending improved surveillance and reporting systems in hospitals while highlighting the need for advancements in diagnostics and vaccines [75]. Current treatments are primarily supportive and expensive, necessitating innovative vector control techniques, especially owing to emerging insecticide resistance. A study in Mumbai identified areas for dengue control improvement, emphasizing accurate case reporting, practical electronic systems for data clarity, and improved diagnostics [76]. Vector surveillance and monitoring of breeding sites are crucial, with innovative mosquito traps, such as Box Passive Traps, suggested for enhanced collection. Integration of molecular epidemiology and community engagement is essential for targeted interventions. In Delhi, a study on health education interventions showed significant increases in knowledge about dengue symptoms and mosquito behaviors among impoverished populations, reinforcing the need for community involvement and socioeconomic approaches to public health strategies [77].

A quasi-experimental study in Sanjay neighborhood over 14 months assessed an intervention program aimed at improving housing conditions and community participation among 314 economically disadvantaged participants. The program resulted in significant enhancements in knowledge and preventive behaviors related to mosquito-borne diseases after two educational sessions, that increased the understanding of symptoms and mosquito behaviors [78]. Furthermore, a mathematical model of dengue transmission underscored the importance of active case finding and highlighted factors such as mosquito biting rates and hospitalization reporting [79]. Furthermore, a multicenter trial conducted from October 2007 to June 2012 evaluated the efficacy of attracticide C21 for *Aedes* mosquito control across Delhi, Bangalore, and Alappuzha. The results showed significant reductions in egg and larvae positivity rates in intervention containers, which demonstrate the potential of attracticide C21 for effective dengue and chikungunya vector surveillance and control [80].

Furthermore, several city-level and regional interventions have provided practical lessons for large-scale control. One of the most cited classical examples comes from Chennai, where Arunachalam et al. implemented an eco-health approach that linked environmental management, community participation, and intersectoral collaboration. The project mobilized local women’s self-help groups, school students, and municipal health workers to eliminate domestic breeding sites and improve solid waste management. Within two years, *Aedes* larval indices decreased by more than 80%, and the intervention areas exhibited a significant reduction in reported dengue cases compared to control zones. Importantly, the study demonstrated that community empowerment and locally tailored environmental actions can sustain vector control even after the withdrawal of external support, offering a scalable model for other urban centers in India and Southeast Asia [81].

#### Pakistan

Dengue in Pakistan has medium–high endemic levels, with an outbreak starting in 1994 and an estimated annual case count of fewer than 40,000 [82]. Control policies in Pakistan emphasize improved sanitation, drainage systems, and environmental management as integral to managing vector-borne diseases. The WHO promotes Integrated Vector Management to sustainably reduce vector populations and human–vector contact. Actions include enhancing sanitation and drainage to minimize breeding sites, particularly in densely populated urban centers. Personal prevention techniques, such as using repellents and protective clothing, are essential but may be impractical because of low income and literacy levels. Effective communication of health messages and training of community health workers is critical for risk reduction. Moreover, strengthening vector and case surveillance, enhancing private-sector healthcare management, and establishing a robust digital information system are necessary. Collaboration across sectors and sufficient resource allocation will further improve dengue control efforts in Pakistan [83].

A study conducted in the Punjab province, Pakistan, focused on reducing dengue incidence through enhanced *Aedes* surveillance and control [84]. This research surveyed 42 councils across four cities, inspecting both natural and artificial habitats with standing water. District health department teams carried out house-to-house surveillance for *Aedes* species and dengue cases, with the data validated and uploaded to the Dengue Tracking System. The analysis revealed that many dengue cases occurred in individuals with recent travel outside the province, while specific habitats, such as water coolers and tire shops, contributed to nearly 30% of the *Aedes* larvae. Temperature variation accounted for 45% of the differences in the number of *Aedes* larvae. The implementation of dengue prevention programs led to a 50% decrease in positive *Aedes* containers and a 70% reduction in reported dengue cases compared with pre-intervention levels (2019–2020). Concurrently, Pakistan faces the dual COVID-19 and dengue epidemics. Strategies involved epidemiological research, diagnostics, and preventative public health measures [84, 85]. Furthermore, studies on the use of Wolbachia bacteria showed effective inhibition of *Aedes* reproduction, although it slightly reduced the female lifespan and fecundity [86].

### Middle East and Australasia

#### Oman

*Ae. aegypti* and *Ae. albopictus* mosquito species have been detected since 2008, but local dengue transmission was only confirmed during outbreaks in November 2018 and January 2019 [87]. Initial cases indicated the first local transmission arising from inadequate vector control measures, weak surveillance, poorly trained staff, and insufficient funding [87]. Factors contributing to outbreaks include artificial habitats for mosquito breeding, increased travel, and importation of goods from endemic regions. Vector control remains critical, relying on reducing human–vector interactions and strengthening entomological surveillance [87]. A successful mosquito elimination campaign was conducted in January 2019 using a combination of thermal fogging with pyrethroid insecticides, larvicidal treatment of breeding sites, environmental sanitation, and intensive door-to-door inspections in Muscat and surrounding districts. This integrated approach led to a rapid decline in adult mosquito indices and successfully interrupted local dengue transmission in the affected areas [88]. Furthermore, an outbreak investigation in Muscat from March to April 2022 confirmed the presence of *Ae. aegypti* larvae and advocated the use of geographic information systems for outbreak prediction and routine vector surveillance, alongside the innovative use of Wolbachia-infected mosquitoes [89].

#### Australia

Effective control measures include pyrethroid spraying and biological methods using predatory copepods. The release of Wolbachia-infected mosquitoes, which can block dengue transmission, holds promise for reducing the disease burden. Furthermore, Rapid Surveillance for Vector Presence systems bolster monitoring and quality assurance in vector control during outbreaks [90, 91].

The management of *Ae. albopictus* in Australia has evolved from a control-focused strategy to a quarantine approach in 2009 due to challenges such as insecticide application gaps and mosquito reintroduction. By implementing pyrethroid spraying on vegetation in early 2011, targeting adult resting sites, *Aedes* populations were drastically reduced, achieving undetectable levels in 70–90% of the surveys by 2015–2016. The key to this success was a surveillance system that confirmed the absence of the *Aedes* establishment in mainland Australia [92]. From 2014 to 2019, Wolbachia-infected mosquitoes were introduced in northern Queensland, leading to a 65% reduction in predicted dengue incidence during interventions and over 95% after two years [93]. Furthermore, studies have indicated that temperature and humidity significantly influence vector abundance, aiding predictive modeling for dengue control efforts [94].

In Australia, a method of *Aedes* control has been targeted insecticidal residual spraying (indoor and harborage spraying) of resting surfaces, especially in outbreak settings and high-risk dwellings. According to national dengue-control guidelines, indoor residual spraying with synthetic pyrethroid insecticides (e.g., deltamethrin or bifenthrin) is applied to common *Aedes aegypti* resting sites (e.g., underside of furniture, dark corners, and closets) following the detection of viremic cases or during outbreaks [95]. Evidence from a major outbreak in Cairns, Queensland, showed that combining contact tracing with targeted indoor residual spraying of likely exposure locations reduced dengue transmission risk by 86–96% compared to unsprayed premises [96]. This targeted indoor residual spraying approach, spraying prioritized indoor resting surfaces rather than blanket walls, has been proposed as more efficient for *Aedes* control because it matches the mosquito’s resting behavior [97]. Moreover, harborage/barrier spraying of outdoor resting or vegetation-adjacent surfaces has been used successfully, for example, to suppress *Aedes albopictus* in fringe/bushland zones or vegetation around dwellings (in the Torres Strait) alongside larval source-reduction efforts [95].

#### Iran

Preventive measures included vector control, public awareness programs, and treatment preparedness [98]. A study on tire trade and its correlation with mosquito surveillance revealed that approximately one-third of Iran’s tire demand is imported from 15 countries where *Ae. aegypti* and *Ae. albopictus* are endemic. Tires, which are often imported unpackaged through unofficial markets, are primarily stored outdoors in urban areas. Such conditions could facilitate the introduction of invasive mosquito species, prompting calls for stricter regulations and improved surveillance at key entry points [98]. Furthermore, a separate study in Guilan Province focused on the entomological surveillance of mosquito populations from May 2018 to December 2019, employing various trapping methods. Of the 3964 samples, 682 were identified as *Aedes* species, particularly *Aedes vexans* and *Aedes geniculatus*, with *Aedes vexans* showing population peaks in October. Although no *Ae. aegypti* or *Ae. albopictus* were found, the findings help in understanding native species, which is essential for future arbovirus control strategies if these invasive species are detected [99].

In West Azerbaijan Province, Iran, a study examined the predictors of dengue prevention practices among 405 health professionals using an online questionnaire. The results indicated that awareness of appropriate preventive actions strongly influenced practices, with beliefs about precautionary effectiveness and difficulty significantly correlated. The highest belief scores related to the severity of dengue risk underscored the need for targeted interventions [99]. Another study explored Drosophila melanogaster as a cost-effective model for biological control of *Aedes* mosquitoes. Samples from 25 counties were identified, revealing the presence of colonies at 23 locations. This study outlines biological rearing methods essential for vector control research [100]. A third study used climatic and topographic data to predict the establishment of *Ae. albopictus* in southeastern Iran. It employs the Analytic Hierarchy Process, emphasizing land use, temperature, elevation, and rainfall as key predictive factors. Geographic mapping indicated that southern areas are high-risk zones for mosquito establishment [101]. Moreover, a field study in southeastern Iran evaluated mosquito traps in urban and rural settings. Despite the previous identification of *Ae. albopictus*, no samples were collected in this study, highlighting the need for ongoing surveillance to confirm vector presence [102].

Timely diagnosis and risk classification of severe diseases, particularly DF, are essential for effective disease management. A study in East Azerbaijan Province, Iran, assessed the knowledge, attitudes, and practices of 948 health care providers (68.5% women) using a structured questionnaire from May to July 2022. Results indicated inadequate overall knowledge (80.4%) with significant differences in attitude scores between physicians and other providers, highlighting the urgent need for educational and training programs [103]. Another study in Mazandaran Province conducted from August 2021 to March 2022 used an online questionnaire to evaluate healthcare workers’ knowledge and practices regarding dengue. Although the majority (83.8%) reported awareness of dengue, knowledge of its symptoms (52%) was notably lower than knowledge of prevention (69%) and transmission (72.2%). Good attitudes (81%) and practices (73%) were reported, but only 49.6% demonstrated good practices related to local transmission [104]. Comprehensive strategies to combat dengue include protective measures, vector elimination, and treatment preparedness. Educating the public and implementing effective insecticide use are crucial for reducing mosquito populations and managing patient care [105].

### Latin America

#### Brazil

Following the return of *Ae. aegypti* mosquitoes, the first outbreak occurred in Roraima between 1981 and 1982, accompanied by major epidemics, such as that in Rio de Janeiro in 1986 [106]. Public health authorities initially focused on educational campaigns and community engagement to curb mosquito populations; however, these strategies only offered short-term solutions. Long-term prevention required extensive sanitation improvements in impoverished neighborhoods, which were not a priority for Brazilian authorities. In 1985, the Pan American Health Organization declared the goal of eradicating *Aedes* unrealistically, advocating sustainable control methods through community participation and diverse control strategies. A critical shift occurred after a severe outbreak in Ribeirão Preto in 1990, which led local authorities to implement comprehensive control measures involving media communication and community involvement. While these efforts have led to some behavioral changes regarding mosquito breeding sites, continued vigilance is necessary because of rising cases linked to increased virus circulation across Brazil [107, 108].

In 1990, Brazil initiated the “*Ae. aegypti* Eradication Program” under the National Health Foundation, which ultimately failed because of coordination issues. This led to the “Intensified Dengue Control Program” in 2001, culminating in the “National Dengue Control Program” of 2002. The program aimed to reduce infestation and dengue cases through epidemiological surveillance, vector control, health education, community mobilization, and political support [109]. Despite these efforts, *Ae. aegypti* populations have developed resistance to temephos, particularly in the northern and southeastern regions [110]. New strategies, including selective monitoring and the use of biological control agents, have been explored. A mathematical model indicated challenges with Wolbachia and sterilization methods, calling for strategic combinations [111]. The integration of Family Health and dengue control programs enhanced community participation in Rio Preto [112]. The release of Wolbachia-infected mosquitoes in Niterói significantly reduced the incidence of dengue, chikungunya, and Zika [113], compared with control areas without releases. A study from August 2017 to December 2019 reported 67 million Wolbachia-infected mosquitoes, achieving a 38% reduction in arboviral diseases [114]. Furthermore, the use of pathogenic fungi resulted in over 80% mortality against *Aedes* larvae [115, 116], and sterile male *Aedes* releases resulted in a 97% decrease in dengue incidence [117]. Finally, the TDV-CYD vaccine was found to be effective, especially for individuals with a history of dengue [118].

#### Colombia

DF remains a significant public health issue in Colombia, with a high endemic level since its onset in 1985, and estimated annual cases below 80,000. The country has adopted various control strategies including community engagement and biological control methods. Monitoring programs for *Ae. aegypti* and *Ae. albopictus* have seen improvements; however, existing systems could benefit from refining surveillance approaches, which are often led by national governments but implemented at the local level. Colombia has introduced a web-based geographical monitoring system, SIVIEN AEDES, which enables field entomologists to track vector density and insecticide resistance [119]. New self-laying traps have supplemented traditional measures, while data from SIVIEN AEDES have aided in developing mathematical models to predict vector populations and implement targeted interventions. Biological control strategies in Colombia have included the release of Wolbachia-infected *Ae. aegypti* mosquitoes as part of a population replacement strategy, aiming to reduce dengue transmission by introducing mosquitoes incapable of transmitting the virus. These interventions were conducted in urban areas and have resulted in significant reductions in dengue incidence. Monitoring data confirmed the establishment of Wolbachia in local mosquito populations, though further evaluation is ongoing to assess long-term efficacy and scalability [120]. Furthermore, autocidal ovitraps were evaluated for their effectiveness in reducing adult *Aedes* mosquito density, demonstrating over 80% reduction efficacy compared to baseline population levels [121]. Innovative communication tools such as WhatsApp have been integrated to enhance community engagement in dengue prevention efforts, demonstrating good user acceptance and improvement in local knowledge about arboviral diseases [122].

#### Peru

Lima had a particularly high incidence rate [123]. In response, the Peruvian Ministry of Health, supported by regional and international partners, implemented a strategy encompassing intensified larvicidal treatments, insecticide spraying, and the establishment of clinical surveillance units to effectively manage outbreaks. Hospitals also set up triage tents for febrile patients and provided nationwide training to physicians [123]. The dengue season coincided with the COVID-19 pandemic, complicating diagnosis due to symptom overlap. Health authorities have focused on resuming vector control and water sanitation efforts, coupled with public education, to address behaviors contributing to mosquito breeding [124]. Innovative mobile technology has been used to enhance preventive behaviors within households, resulting in a notable reduction in dengue transmission risk [125]. Moreover, a randomized controlled trial assessing spatial repellents showed a 34% reduction in mosquito-borne viral infections, decreasing *Ae. aegypti* populations by 28.6% and blood-feeding rates by 12.4% [126].

#### Paraguay

DF is a significant public health concern in Paraguay and is categorized as highly endemic, with its emergence in 1992. A dramatic surge in the number of DF cases was recorded, escalating from 12,497 in 2023 to over 240,000 in 2024, marking an increase of more than 1825% [127]. This escalation necessitated urgent measures, including enhancement of surveillance systems and vector control programs, along with robust public health campaigns to curb the outbreak [127]. In response, the TopaDengue project was initiated as a community-based intervention in the vulnerable areas of Asunción, where the prevalence of *Ae. aegypti* larvae reached 20%. Local volunteers regularly conduct home inspections to identify mosquito breeding sites and classify containers into active, potential, or protected containers based on their status for breeding [128]. An innovative tele-epidemiological surveillance system has been established that automates case reporting through phone calls, allowing immediate case prioritization and follow-up by community health agents. From April 2010 to August 2011, the system recorded numerous suspected DF cases and confirmed positive results in 82% of the tested samples. This cost-effective approach shows promise for broader applications in managing dengue and other public health concerns in Paraguay, highlighting the importance of community engagement and technological integration in disease control strategies [129].

### Europe

#### France

Dengue in France shows distinct epidemiological patterns between mainland France and its tropical territories. In Réunion Island, dengue became established in the late 1990s to early 2000s and has shown clear endemic trends, with outbreaks escalating since 2018. Severe cases increased from 0.4% in 2018 to 0.8% in 2021, alongside more pediatric infections (from 8 to 15%) and atypical forms, including 108 post-dengue maculopathy cases. These findings prompted health authorities to adapt public health strategies by accounting for comorbidities and long-term management in tropical contexts [130]. In contrast, mainland France has historically reported imported cases but has recently seen autochthonous transmission, reflecting changing climatic and environmental conditions favorable to *Aedes albopictus*. In 2023, a small cluster of three locally acquired dengue cases was confirmed in northern France (Île-de-France region), signaling a growing risk in temperate zones. Authorities immediately launched case-finding campaigns, public awareness programs, and door-to-door surveys within a 150-m radius. Two deltamethrin treatments were conducted to prevent further transmission, coordinated with regional health agencies [131]. Studies from southern France further show that ultralow-volume insecticide spraying using handheld thermal foggers yields high mosquito mortality, while vehicle-mounted methods are less effective, which highlights the need for locally adapted interventions [132].

#### Portugal

Dengue in Portugal has been classified as a low endemic, with autochthonous cases first confirmed in 2012, particularly in Madeira, where two initial cases were reported on October 3, 2012 [133]. Testing revealed that the serotype was dengue virus serotype 1. Following an outbreak that resulted in 1,891 cases by November 25, health authorities took swift action to curb the spread of the virus [133]. Enhanced control measures ensued at Madeira Airport, including disinfection of all departing flights and reinforced mosquito surveillance at various transport hubs, particularly cruise ship ports. Public awareness campaigns have also ensued, employing community involvement to promote personal protective measures and the removal of breeding sites [133]. Recommendations for health professionals regarding diagnostic and management protocols for DF have been established. Blood safety protocols included quarantining blood samples and screening donors for dengue virus using RT-PCR. Despite these actions, current vector control methods remain insufficient, suggesting the need for innovative and immediately implementable solutions. Research has highlighted the auto-dissemination of pyriproxyfen as a potential larvicidal technique, showing moderate success in reducing mosquito populations through the strategic placement of traps, but efficacy varied significantly based on local conditions and mosquito density, indicating that further investigation is essential [134].

#### Spain

The origins of the *Aedes* mosquito in Spain remain uncertain; however, significant populations are established in Mediterranean France, particularly *Ae. albopictus*, which was first documented in Spain in 2004. Following this discovery, the Spanish Ministry of Health prioritized surveillance, although initial monitoring in used tire storage facilities reported no presence. Vector control programs across Spain lack consistency, with municipalities frequently outsourcing control measures with varying degrees of efficacy. Catalonia exemplifies integrated vector management through community-based outreach, larvicide application, and adulticide use, resulting in reductions in breeding sites. An extensive campaign from 2008 to 2010 utilized house visits to educate communities about breeding habitats. A study evaluating a multifaceted intervention strategy demonstrated significant reductions in mosquito egg counts, emphasizing homeowner cooperation as crucial to success [135, 136].

In Spain, biological control measures against the invasive mosquito species *Ae. albopictus* have begun, particularly through the establishment of Wolbachia infections in wild populations. A study demonstrated that hybrid males, produced via hybridization, effectively sterilized 99.9% of wild female *Ae. albopictus* eggs without prior treatment, marking a significant advance in control methods for this vector of dengue, chikungunya, and Zika viruses [137]. Furthermore, research in Valencia found that 94% of mosquito samples tested were naturally infected with Wolbachia [138]. To complement these efforts, the SIT was introduced as an environmentally friendly control option. A pilot project by the Valencia Regional Agriculture Department produced over 15 million sterile *Ae. albopictus* males from 2018 to 2020, which was applied to more than 80 hectares. The project followed WHO guidelines for quality control in mosquito rearing, achieving effective male pupae recovery and contributing to the development of medium-scale mosquito production strategies [139].

### North America

#### United States

In 2020, 72 locally acquired dengue cases were reported in Key Largo, prompting an operational response that included increased manpower and enhanced vector control measures utilizing long-lasting larvicides and ultralow-volume spraying to manage *Ae. aegypti* populations [140]. This response effectively controlled the mosquito population, and no new cases have occurred since October 2020 [141]. In recent years, integrated vector management strategies have evolved. Care2In mosquito traps target both adult and immature mosquitoes, successful in reducing their populations by disseminating the larvicide pyriproxyfen and fungal spores that impair survival [140]. Furthermore, the release of Wolbachia-infected male *Ae. aegypti* mosquitoes in Houston resulted in a remarkable 93% reduction in wild populations [142]. These Wolbachia males also had significantly lowered egg counts, indicating the potential for integrated vector management [143]. In biological control efforts, Utricularia macrorhiza demonstrated high larvicidal efficacy against *Aedes* mosquitoes, achieving 100% and 95% mortality in larvae in trials, showcasing its potential as an effective mosquito control method [144].

## Discussion

We identified best practices for managing and controlling *Aedes* mosquitoes and the viral pathogens they transmit, as utilized in the selected countries. Advanced control strategies incorporate predictive modeling, community engagement, and innovative vector management, such as the use of Wolbachia-infected mosquitoes and new surveillance technologies. Despite successes, rising cases necessitate enhanced monitoring, public education, and comprehensive intervention tactics.

Given the rising threat of DF and potential outbreaks globally, these recommendations and control measures are effective strategies for managing the disease. In countries with a heightened risk of transmission, implementing comparable strategies is crucial. Biological interventions using Wolbachia-infected mosquitoes have significantly reduced dengue transmission, with Singapore reporting a 77% decline in cases through large-scale releases of the wMel strain, which leverages cytoplasmic incompatibility to suppress wild mosquito populations [63]. Indonesia observed an 83% reduction in severe dengue hemorrhagic fever cases in Yogyakarta, attributed to Wolbachia’s activation of mosquito immune pathways and competition for host lipids, which inhibit viral replication [145]. In Brazil, integrated approaches combining Wolbachia deployments with larvicidal traps reduced arboviral diseases by 38%, though scalability remains challenged by insecticide resistance [146]. Success in urban settings like Singapore relies on centralized infrastructure for Wolbachia density monitoring, while rural regions like Jaffna, Sri Lanka, capitalize on pre-existing Wolbachia prevalence in local Culex mosquitoes. However, ecological and social barriers persist: high temperatures (> 35°C) destabilize Wolbachia in arid climates [120], and community hesitancy in Malaysia underscores the need for culturally adapted education. Innovations such as hybrid wMel-wAlbB strains show promise for thermal resilience, while genetic engineering to amplify immune effectors could enhance viral blocking. Long-term cost–benefit analyses in Brazil project $1.3 billion in savings over 20 years, which emphasizes the importance of fiscal planning for global scalability [146].

Similarly, SIT showed promise in Spain and Sri Lanka but requires advanced rearing facilities and long-term funding, which limits its feasibility in resource-limited settings like Paraguay. Key mechanisms underpinning SIT, such as precise sex-separation systems to minimize female releases and optimized sterilization protocols balancing sterility with male competitiveness, require advanced infrastructure, including irradiation facilities and large-scale rearing laboratories, which are often prohibitive [147, 148]. Long-term funding challenges further complicate implementation, as seen in Reunion Island, where multi-year delays arose from budget constraints [149]. Additionally, regulatory hurdles and stakeholder engagement demands, exemplified by Brazil’s two-year authorization process, highlight systemic bottlenecks [150]. Innovative hybrid approaches, such as combining SIT with incompatible insect techniques to reduce female contamination risks (the proportion of females infected with Wolbachia among the released mosquito population), as demonstrated in China with a 94% population decline, offer potential solutions [151]. For Paraguay, phased strategies prioritizing small-scale pilot trials, international partnerships for technology transfer, and integration with existing vector control programs could mitigate barriers while building local capacity [152].

Alongside the outlined guidelines, certain targeted actions could prove beneficial. Key strategies include distributing mosquito nets, forming specialized task forces at the provincial level, and conducting entomological monitoring to reduce transmission [69]. However, barriers, such as inadequate funding, lack of effective surveillance, and cultural obstacles, challenge implementation. To overcome these issues, cross-sector collaboration is essential, along with enhancing local capabilities and educating communities. Community-based vector control programs can empower residents to identify and eliminate mosquito breeding sites. Integrating climate data and predictive models into surveillance systems can improve early warning and preventive measures. Strengthening partnerships with international organizations can facilitate knowledge sharing and resource allocation. Some countries have employed advanced technologies, such as biological methods, to control mosquito populations, necessitating proper infrastructure and resource investment. Improvements in diagnostic laboratory capabilities and ongoing training for healthcare personnel are also critical.

To improve the understanding of DF prevalence, health authorities worldwide must conduct nationwide surveys to assess the distribution of dengue serotypes. These surveys should involve vector surveillance and virus identification tests to identify both serotypes and their corresponding vectors accurately. Subsequently, dengue should be designated as a reportable disease, and an integrated dengue surveillance system linked to the national health information system should be established, including key indicators for ongoing monitoring. Additionally, developing outbreak preparedness plans that engage relevant agencies is vital. National strategies for dengue control must be strengthened, as the disease is often misdiagnosed as malaria and remains overlooked as a neglected tropical disease. Emphasis on vector control and public education regarding methods to decrease mosquito bites, such as wearing protective clothing, is essential. Source reduction should be prioritized through environmental modifications like covering water containers and disposing of unnecessary items properly. While insecticides such as organophosphates (e.g., temephos) and pyrethroids are beneficial, resistance among *Aedes aegypti* and *Aedes albopictus* has been reported. Innovative techniques, such as genetically modified mosquitoes and Wolbachia-based interventions, promise to drastically reduce disease-carrying vector populations, potentially by up to 95% [153]. These methods include "population replacement", [154], in which either genetically modified or Wolbachia-infected mosquitoes, incapable of transmitting pathogens, replace susceptible wild populations [155]. The effective deployment of these strategies could impact the transmission rates of other diseases, including malaria, yellow fever, Zika, and chikungunya, enhancing public health outcomes and reducing the burden of vector-borne diseases.

Countries like Brazil and Paraguay have implemented extensive educational programs to raise awareness and provide training on dengue control and prevention. These programs include education on preventing mosquito bites, environmental improvement, and self-care measures. Regular training for the public and healthcare personnel has reduced disease outbreaks. Some countries use geographic monitoring systems to identify and control high-risk areas. These systems collect and analyze geographic and epidemiological data to identify high-risk areas and implement targeted control measures. This approach is effective in regions with large populations or new areas at risk of epidemic outbreaks. Brazil has been active in environmental improvement and waste management. This involves eliminating stagnant water sources and collecting solid waste related to controlling vector mosquitoes. These actions reduce mosquito breeding sites and consequently decrease infectious disease outbreaks.

Countries facing the threat of febrile illnesses, including DF, should enhance their diagnostic capabilities and commitment to accurate disease identification. Establishing laboratory surveillance for dengue is vital to distinguish it from similar clinical conditions like malaria, yellow fever, and chikungunya [156]. An advancement would be implementing NS1 dipstick testing at primary healthcare facilities, as it is user-friendly, sensitive, and delivers rapid results. Training frontline health workers and emphasizing dengue as a primary fever cause is essential for effective management [157]. Furthermore, this initiative should extend to laboratory scientists and entomologists to bolster accurate dengue diagnosis and improve vector control measures. Given the ongoing concerns regarding the transmission of Zika from the Americas to other parts of the world, countries must remain vigilant for cases of Zika virus disease [158], particularly those located within the dengue belt where *Ae. aegypti* and *Ae. albopictus* mosquitoes are prevalent. Implementing effective surveillance and monitoring for Zika cases and appropriate measures for controlling mosquito populations are essential. Since intrauterine transmission, sexual contact, and blood-borne transmission of Zika have been confirmed, it is important to promote suitable precautions within communities to mitigate the risk of infection.

To optimize the integration of *Aedes* control strategies into national guidelines, policymakers should prioritize adaptive frameworks that allow regions to tailor interventions to local ecological, socioeconomic, and climatic contexts. This includes fostering intersectoral collaboration (health, environmental, and urban planning sectors) to address root causes of outbreaks, investing in community-driven solutions such as mobile surveillance tools and education campaigns, and leveraging global knowledge-sharing platforms to disseminate best practices for Wolbachia deployments, vaccine equity, and insecticide resistance management.

Comparing strategies between endemic and epidemic countries reveals that control approaches are shaped by both resource availability and entomological context. In endemic regions such as Indonesia, Brazil, and Sri Lanka, long-term integrated programs emphasize sustained community engagement, Wolbachia-based vector replacement, and continuous surveillance, supported by dedicated public health infrastructure. For example, the World Mosquito Program in Yogyakarta, Indonesia, reported about 77% reduction in dengue incidence after releasing Wolbachia-infected mosquitoes following two years of community engagement and extensive monitoring. In contrast, epidemic-prone or newly affected countries, like France, Spain, and Oman, tend to focus on rapid response mechanisms, outbreak forecasting, and emergency vector control operations. In Oman, the outbreak of autochthonous dengue in Muscat Governorate in 2018–2019 was contained through fast surveillance, public awareness/media campaigns, extensive vector surveys, elimination of breeding sites, and strong coordination of case finding efforts [88].

Resource availability plays a role in implementation as wealthier nations often invest in predictive modeling, advanced diagnostic systems, and insecticide resistance monitoring, while lower- and middle-income countries rely more on community-driven initiatives and international collaborations. However, success across all contexts depends on aligning interventions with local ecological and entomological conditions, rather than solely on economic capacity.

A major challenge that continues to undermine vector control programs is the growing resistance of Aedes mosquitoes to commonly used insecticides. Resistance to organophosphates such as temephos and pyrethroids has been widely documented in regions with long-term chemical control, including Brazil (In Ceará: RR up to about 192.7 for temephos; resistance also to cypermethrin) [159], Southeast Asia (Malaysia, Indonesia, etc.) [160], and parts of Africa (e.g., Gabon) [161]. Studies in Brazil reported that Ae. aegypti populations from the northern and northeastern regions have developed high levels of resistance to temephos, significantly reducing larvicide effectiveness (spatial–temporal surveys) [162]. Similar patterns have been seen in Asia, where pyrethroid resistance (e.g., mutations V1016G, F1534C, S989P) threatens indoor residual spraying and space-spray programs [160]. This trend highlights the urgent need for continuous insecticide resistance monitoring and integration of non-chemical strategies, such as Wolbachia-based approaches, SITs, and environmental management, to sustain long-term vector control effectiveness.

A comparative synthesis of dengue control research across 21 countries reveals that no single approach can fully control *Aedes*-borne diseases, but combining scientific innovation with community participation and sustained political commitment offers the best outcomes. Among the most studied interventions, Wolbachia-based population replacement stands out for its robust field evidence. Trials in Singapore, Indonesia, Brazil, and Australia consistently showed reductions of 70–83% in dengue incidence relative to baseline or control areas prior to intervention after large-scale releases of Wolbachia-infected *Aedes aegypti*. These results confirm the biological feasibility and epidemiological impact of endosymbiont-based vector control. However, some studies reported reduced efficacy in regions with extreme temperatures and high rainfall variability, highlighting the need for ongoing genomic adaptation and modeling research to ensure stability under diverse ecological conditions.

Another promising innovation is the SIT, evaluated in Sri Lanka, Spain, and parts of India, which demonstrated substantial suppression of local *Aedes* populations through the mass release of sterilized males. While technically effective, SIT remains operationally demanding and expensive due to the need for continuous mosquito production, irradiation facilities, and precise release logistics. These limitations have prompted hybrid approaches combining SIT with Wolbachia or chemical attractants to improve scalability and cost-efficiency. Predictive surveillance and data-driven modeling approaches have also evolved rapidly. In Taiwan and China, integrating meteorological data, entomological indices, and case notifications into machine learning algorithms improved outbreak prediction accuracy by up to 25–40%. Such models provide early warnings that guide resource allocation, yet their performance depends heavily on the quality and granularity of input data, which is often inconsistent in low-resource settings.

Community engagement and eco-health interventions remain the cornerstone of sustainable dengue control. Classic studies from Chennai, India, and major Brazilian cities have shown that structured community participation through neighborhood cleanup campaigns, household source reduction, and behavior-change education—can reduce larval indices by more than 70% while fostering long-term awareness. However, maintaining public motivation beyond the project period is a persistent challenge, and the success of these interventions largely depends on social trust, gender inclusion, and municipal coordination.

Chemical and biological control methods, such as larviciding, adulticiding, and IVM continue to play complementary roles, particularly during outbreaks. Yet, increasing insecticide resistance, environmental toxicity, and financial burden have limited their sustainability. Recent programs in Oman and the Philippines suggest that combining targeted fogging with environmental management and real-time geographic information system surveillance significantly improves impact while minimizing chemical use. The cross-country evidence indicates that integrated, research-informed, and community-supported strategies yield the most durable results. Countries that have successfully reduced dengue incidence such as Singapore, Indonesia, and Malaysia share three features: (1) continuous entomological surveillance and early-warning systems; (2) incorporation of new biological and digital tools within national frameworks; and (3) active community involvement backed by political and financial commitment. Moving forward, the key research priorities include evaluating long-term Wolbachia dynamics under climate change, scaling up digital surveillance models for data-poor regions, and developing cost-effective, socially acceptable strategies for sustained vector control.

This study had limitations that should be noted and considered. One limitation is that our study may not provide precise results regarding dengue control measures’ economic and social impact. Additionally, the available data are limited to certain countries and regions, and future changes in the patterns of disease outbreaks may affect the accuracy of these findings. The scope of this review focuses primarily on best practices for the management and control of *Aedes* mosquitoes, which may not encompass all relevant strategies in broader vector control initiatives. Furthermore, relying on published literature means that successful anecdotal practices or innovative grassroots strategies might have been overlooked. It is also important to note that only some of the main and selected methods implemented in the countries have been mentioned here, and this does not imply that those countries have not used methods or policies not included in this study. Our study includes countries with high dengue incidence and mortality rates, enhancing findings’ generalizability across various regions. It emphasizes community engagement, innovative control methods, and ongoing research, reflecting successful trends in dengue management. By synthesizing information from diverse nations, the study identifies transferable strategies that can be adapted to different socio-cultural contexts, providing valuable insights for effective dengue control and prevention. Moreover, although this review included 21 countries across several WHO regions, data availability was not globally consistent. Some high-burden regions, particularly in Africa, were underrepresented because of limited access to systematically reported outcomes in indexed publications. This underrepresentation reflects a documentation gap rather than a lack of vector control efforts. Strengthening surveillance systems, operational evidence, and scientific reporting in these settings is essential to enhance future global comparisons. The included countries mainly represent settings with established evidence on dengue incidence and mortality, which supports the broader applicability of the main practices identified, especially those emphasizing community engagement, integrated surveillance, and innovative mosquito control tools. Continued improvement in data reporting may enable a more comprehensive representation of global best practices in dengue prevention and *Aedes* control.

## Conclusions

Our findings indicate that successful dengue prevention relies on the integration of robust surveillance, active community engagement, and innovative biological and technological approaches. National programs that implement the IVM framework, through cross-sector collaboration, adaptive policies, and sustained public participation, consistently achieve stronger outcomes. The results also underscore the importance of tailoring strategies to the ecological, social, and economic characteristics of each region while maintaining coordination at broader levels. Strengthening institutional capacity, ensuring policy continuity, and promoting community-driven initiatives are critical for sustaining long-term success. These insights can guide policymakers and public health leaders in developing resilient, inclusive, and adaptive vector management systems capable of addressing both current and emerging mosquito-borne disease threats.

## Supplementary Information

Supplementary material 1.

## Data Availability

No datasets were generated or analysed during the current study.
