# Supplementary material for: Managing and controlling diseases transmitted by Aedes mosquitoes: a review on best practices
Source: Trop Med Health. 2026 Jan 9;54:12. doi: 10.1186/s41182-025-00890-7 (PMC12797487; doi:10.1186/s41182-025-00890-7)
Supplement: Supplementary file 1 — Supplementary material 1. [file 41182_2025_890_MOESM1_ESM.docx]

**Table S1.** Search strategies, search dates, and number of PubMed electronic database results for Taiwan, India, Oman, Singapore, Malaysia, Sri Lanka, Indonesia, Pakistan, China, Philippines, Japan, Brazil, Paraguay, Peru, United States, Colombia, France, Portugal, Spain, Australia, and Iran.

| **Step** | **Search strategy** | **Results** |
| --- | --- | --- |
| **Taiwan (Search date: August 25, 2024)** | | |
| 1 | "Dengue"[Mesh] OR "Arbovirus Infections"[Mesh] OR "Aedes"[Mesh] OR "Severe Dengue"[Mesh] OR "Severe Dengue"[Title/Abstract] OR "Aedes"[Title/Abstract] OR "Dengue"[Title/Abstract] OR "Arbovirus Infection"[Title/Abstract] OR "Dengue Fever"[Title/Abstract] OR "Classical Dengue"[Title/Abstract] OR "Break-Bone Fever"[Title/Abstract] OR "Break Bone Fever"[Title/Abstract] OR "Breakbone Fever"[Title/Abstract] OR "Dengue Hemorrhagic Fever"[Title/Abstract] OR "Singapore Hemorrhagic Fever"[Title/Abstract] OR "Thai Hemorrhagic Fever"[Title/Abstract] OR "Philippine Hemorrhagic Fever"[Title/Abstract] OR "Dengue Shock Syndrome"[Title/Abstract] OR "Mosquito-borne Flavivirus"[Title/Abstract] OR "Dengue Outbreak"[Title/Abstract] OR "Aedes Aegypti"[Title/Abstract] OR "Aedes Albopictus"[Title/Abstract] | 81,181 |
| 2 | "Taiwan"[Mesh] OR "Taiwan"[Title/Abstract] OR "Formosa"[Title/Abstract] OR "Taiwanese"[Title/Abstract] OR Taipei[Title/Abstract] | 72,577 |
| 3 | #1 AND #2 | 512 |
| **India (Search date: August 26, 2024)** | | |
| 1 | "Dengue"[Mesh] OR "Arbovirus Infections"[Mesh] OR "Aedes"[Mesh] OR "Severe Dengue"[Mesh] OR "Severe Dengue"[Title/Abstract] OR "Aedes"[Title/Abstract] OR "Dengue"[Title/Abstract] OR "Arbovirus Infection"[Title/Abstract] OR "Dengue Fever"[Title/Abstract] OR "Classical Dengue"[Title/Abstract] OR "Break-Bone Fever"[Title/Abstract] OR "Break Bone Fever"[Title/Abstract] OR "Breakbone Fever"[Title/Abstract] OR "Dengue Hemorrhagic Fever"[Title/Abstract] OR "Singapore Hemorrhagic Fever"[Title/Abstract] OR "Thai Hemorrhagic Fever"[Title/Abstract] OR "Philippine Hemorrhagic Fever"[Title/Abstract] OR "Dengue Shock Syndrome"[Title/Abstract] OR "Mosquito-borne Flavivirus"[Title/Abstract] OR "Dengue Outbreak"[Title/Abstract] OR "Aedes Aegypti"[Title/Abstract] OR "Aedes Albopictus"[Title/Abstract] | 81,181 |
| 2 | "India"[Mesh] OR "India"[Title/Abstract] OR "Indian"[Title/Abstract] OR "Indians"[Title/Abstract] OR "Bharat"[Title/Abstract] OR "Hindustan"[Title/Abstract] OR "Indian Population"[Title/Abstract] OR "Indian Subcontinent"[Title/Abstract] | 252,493 |
| 3 | #1 AND #2 | 3,458 |
| **Oman** **(Search date: August 26, 2024)** | | |
| 1 | "Dengue"[Mesh] OR "Arbovirus Infections"[Mesh] OR "Aedes"[Mesh] OR "Severe Dengue"[Mesh] OR "Severe Dengue"[Title/Abstract] OR "Aedes"[Title/Abstract] OR "Dengue"[Title/Abstract] OR "Arbovirus Infection"[Title/Abstract] OR "Dengue Fever"[Title/Abstract] OR "Classical Dengue"[Title/Abstract] OR "Break-Bone Fever"[Title/Abstract] OR "Break Bone Fever"[Title/Abstract] OR "Breakbone Fever"[Title/Abstract] OR "Dengue Hemorrhagic Fever"[Title/Abstract] OR "Singapore Hemorrhagic Fever"[Title/Abstract] OR "Thai Hemorrhagic Fever"[Title/Abstract] OR "Philippine Hemorrhagic Fever"[Title/Abstract] OR "Dengue Shock Syndrome"[Title/Abstract] OR "Mosquito-borne Flavivirus"[Title/Abstract] OR "Dengue Outbreak"[Title/Abstract] OR "Aedes Aegypti"[Title/Abstract] OR "Aedes Albopictus"[Title/Abstract] | 81,181 |
| 2 | "Oman"[Mesh] OR Oman[Title/Abstract] OR "Sultanate of Oman"[Title/Abstract] OR "Muscat"[Title/Abstract] | 5,111 |
| 3 | #1 AND #2 | 28 |
| **Singapore** **(Search date: August 26, 2024)** | | |
| 1 | "Dengue"[Mesh] OR "Arbovirus Infections"[Mesh] OR "Aedes"[Mesh] OR "Severe Dengue"[Mesh] OR "Severe Dengue"[Title/Abstract] OR "Aedes"[Title/Abstract] OR "Dengue"[Title/Abstract] OR "Arbovirus Infection"[Title/Abstract] OR "Dengue Fever"[Title/Abstract] OR "Classical Dengue"[Title/Abstract] OR "Break-Bone Fever"[Title/Abstract] OR "Break Bone Fever"[Title/Abstract] OR "Breakbone Fever"[Title/Abstract] OR "Dengue Hemorrhagic Fever"[Title/Abstract] OR "Singapore Hemorrhagic Fever"[Title/Abstract] OR "Thai Hemorrhagic Fever"[Title/Abstract] OR "Philippine Hemorrhagic Fever"[Title/Abstract] OR "Dengue Shock Syndrome"[Title/Abstract] OR "Mosquito-borne Flavivirus"[Title/Abstract] OR "Dengue Outbreak"[Title/Abstract] OR "Aedes Aegypti"[Title/Abstract] OR "Aedes Albopictus"[Title/Abstract] | 81,181 |
| 2 | Singapore[Title/Abstract] OR Singapore[Mesh] OR "Republic of Singapore"[Title/Abstract] | 25,214 |
| 3 | #1 AND #2 | 541 |
| **Malaysia (Search date: August 26, 2024)** | | |
| 1 | "Dengue"[Mesh] OR "Arbovirus Infections"[Mesh] OR "Aedes"[Mesh] OR "Severe Dengue"[Mesh] OR "Severe Dengue"[Title/Abstract] OR "Aedes"[Title/Abstract] OR "Dengue"[Title/Abstract] OR "Arbovirus Infection"[Title/Abstract] OR "Dengue Fever"[Title/Abstract] OR "Classical Dengue"[Title/Abstract] OR "Break-Bone Fever"[Title/Abstract] OR "Break Bone Fever"[Title/Abstract] OR "Breakbone Fever"[Title/Abstract] OR "Dengue Hemorrhagic Fever"[Title/Abstract] OR "Singapore Hemorrhagic Fever"[Title/Abstract] OR "Thai Hemorrhagic Fever"[Title/Abstract] OR "Philippine Hemorrhagic Fever"[Title/Abstract] OR "Dengue Shock Syndrome"[Title/Abstract] OR "Mosquito-borne Flavivirus"[Title/Abstract] OR "Dengue Outbreak"[Title/Abstract] OR "Aedes Aegypti"[Title/Abstract] OR "Aedes Albopictus"[Title/Abstract] | 81,181 |
| 2 | Malaysia[Title/Abstract] OR Malaysia[Mesh] OR "Malaysian"[Title/Abstract] OR "Malaya Federation"[Title/Abstract] OR "Federation of Malaya"[Title/Abstract] OR "Malay Federation"[Title/Abstract] OR "Malaya"[Title/Abstract] OR "Sarawak"[Title/Abstract] "Malay Peninsula"[Title/Abstract] OR Sabah[Title/Abstract] | 1,260 |
| 3 | #1 AND #2 | 31 |
| **Sri Lanka (Search date: August 26, 2024)** | | |
| 1 | "Dengue"[Mesh] OR "Arbovirus Infections"[Mesh] OR "Aedes"[Mesh] OR "Severe Dengue"[Mesh] OR "Severe Dengue"[Title/Abstract] OR "Aedes"[Title/Abstract] OR "Dengue"[Title/Abstract] OR "Arbovirus Infection"[Title/Abstract] OR "Dengue Fever"[Title/Abstract] OR "Classical Dengue"[Title/Abstract] OR "Break-Bone Fever"[Title/Abstract] OR "Break Bone Fever"[Title/Abstract] OR "Breakbone Fever"[Title/Abstract] OR "Dengue Hemorrhagic Fever"[Title/Abstract] OR "Singapore Hemorrhagic Fever"[Title/Abstract] OR "Thai Hemorrhagic Fever"[Title/Abstract] OR "Philippine Hemorrhagic Fever"[Title/Abstract] OR "Dengue Shock Syndrome"[Title/Abstract] OR "Mosquito-borne Flavivirus"[Title/Abstract] OR "Dengue Outbreak"[Title/Abstract] OR "Aedes Aegypti"[Title/Abstract] OR "Aedes Albopictus"[Title/Abstract] | 81,181 |
| 2 | "Sri Lanka"[Title/Abstract] OR "Sri Lanka"[Mesh] OR "Sri Lankan"[Title/Abstract] OR "Ceylon"[Title/Abstract] | 11,915 |
| 3 | #1 AND #2 | 500 |
| **Indonesia (Search date: August 26, 2024)** | | |
| 1 | "Dengue"[Mesh] OR "Arbovirus Infections"[Mesh] OR "Aedes"[Mesh] OR "Severe Dengue"[Mesh] OR "Severe Dengue"[Title/Abstract] OR "Aedes"[Title/Abstract] OR "Dengue"[Title/Abstract] OR "Arbovirus Infection"[Title/Abstract] OR "Dengue Fever"[Title/Abstract] OR "Classical Dengue"[Title/Abstract] OR "Break-Bone Fever"[Title/Abstract] OR "Break Bone Fever"[Title/Abstract] OR "Breakbone Fever"[Title/Abstract] OR "Dengue Hemorrhagic Fever"[Title/Abstract] OR "Singapore Hemorrhagic Fever"[Title/Abstract] OR "Thai Hemorrhagic Fever"[Title/Abstract] OR "Philippine Hemorrhagic Fever"[Title/Abstract] OR "Dengue Shock Syndrome"[Title/Abstract] OR "Mosquito-borne Flavivirus"[Title/Abstract] OR "Dengue Outbreak"[Title/Abstract] OR "Aedes Aegypti"[Title/Abstract] OR "Aedes Albopictus"[Title/Abstract] | 81,181 |
| 2 | Indonesia[Title/Abstract] OR Indonesia[Mesh] OR "Republic of Indonesia"[Title/Abstract] OR "Jakarta"[Title/Abstract] OR "Netherlands East Indies" OR "West Irian"[Title/Abstract] OR "Indonesian New Guinea"[Title/Abstract] OR "Irian Jaya"[Title/Abstract] OR "Timor"[Title/Abstract] OR "Java"[Title/Abstract] OR "Madoera"[Title/Abstract] OR "Madura"[Title/Abstract] OR "Sumatra"[Title/Abstract] OR "Celebes"[Title/Abstract] OR "Sulawesi"[Title/Abstract] OR "Bali"[Title/Abstract] OR "Malay Archipelago"[Title/Abstract] | 31,118 |
| 3 | #1 AND #2 | 724 |
| **Pakistan (Search date: August 26, 2024)** | | |
| 1 | "Dengue"[Mesh] OR "Arbovirus Infections"[Mesh] OR "Aedes"[Mesh] OR "Severe Dengue"[Mesh] OR "Severe Dengue"[Title/Abstract] OR "Aedes"[Title/Abstract] OR "Dengue"[Title/Abstract] OR "Arbovirus Infection"[Title/Abstract] OR "Dengue Fever"[Title/Abstract] OR "Classical Dengue"[Title/Abstract] OR "Break-Bone Fever"[Title/Abstract] OR "Break Bone Fever"[Title/Abstract] OR "Breakbone Fever"[Title/Abstract] OR "Dengue Hemorrhagic Fever"[Title/Abstract] OR "Singapore Hemorrhagic Fever"[Title/Abstract] OR "Thai Hemorrhagic Fever"[Title/Abstract] OR "Philippine Hemorrhagic Fever"[Title/Abstract] OR "Dengue Shock Syndrome"[Title/Abstract] OR "Mosquito-borne Flavivirus"[Title/Abstract] OR "Dengue Outbreak"[Title/Abstract] OR "Aedes Aegypti"[Title/Abstract] OR "Aedes Albopictus"[Title/Abstract] | 81,181 |
| 2 | Pakistan[Title/Abstract] OR Pakistan[Mesh] OR "Islamic Republic of Pakistan"[Title/Abstract] | 37,596 |
| 3 | #1 AND #2 | 552 |
| **China (Search date: August 27, 2024)** | | |
| 1 | "Dengue"[Mesh] OR "Arbovirus Infections"[Mesh] OR "Aedes"[Mesh] OR "Severe Dengue"[Mesh] OR "Severe Dengue"[Title/Abstract] OR "Aedes"[Title/Abstract] OR "Dengue"[Title/Abstract] OR "Arbovirus Infection"[Title/Abstract] OR "Dengue Fever"[Title/Abstract] OR "Classical Dengue"[Title/Abstract] OR "Break-Bone Fever"[Title/Abstract] OR "Break Bone Fever"[Title/Abstract] OR "Breakbone Fever"[Title/Abstract] OR "Dengue Hemorrhagic Fever"[Title/Abstract] OR "Singapore Hemorrhagic Fever"[Title/Abstract] OR "Thai Hemorrhagic Fever"[Title/Abstract] OR "Philippine Hemorrhagic Fever"[Title/Abstract] OR "Dengue Shock Syndrome"[Title/Abstract] OR "Mosquito-borne Flavivirus"[Title/Abstract] OR "Dengue Outbreak"[Title/Abstract] OR "Aedes Aegypti"[Title/Abstract] OR "Aedes Albopictus"[Title/Abstract] | 81,195 |
| 2 | China[Title/Abstract] OR China[Mesh] OR "People's Republic of China"[Title/Abstract] OR "Inner Mongolia"[Title/Abstract] OR "Manchuria"[Title/Abstract] OR "Sinkiang"[Title/Abstract] OR "Mainland China"[Title/Abstract] | 453,878 |
| 3 | #1 AND #2 | 1,666 |
| **Philippines (Search date: August 27, 2024)** | | |
| 1 | "Dengue"[Mesh] OR "Arbovirus Infections"[Mesh] OR "Aedes"[Mesh] OR "Severe Dengue"[Mesh] OR "Severe Dengue"[Title/Abstract] OR "Aedes"[Title/Abstract] OR "Dengue"[Title/Abstract] OR "Arbovirus Infection"[Title/Abstract] OR "Dengue Fever"[Title/Abstract] OR "Classical Dengue"[Title/Abstract] OR "Break-Bone Fever"[Title/Abstract] OR "Break Bone Fever"[Title/Abstract] OR "Breakbone Fever"[Title/Abstract] OR "Dengue Hemorrhagic Fever"[Title/Abstract] OR "Singapore Hemorrhagic Fever"[Title/Abstract] OR "Thai Hemorrhagic Fever"[Title/Abstract] OR "Philippine Hemorrhagic Fever"[Title/Abstract] OR "Dengue Shock Syndrome"[Title/Abstract] OR "Mosquito-borne Flavivirus"[Title/Abstract] OR "Dengue Outbreak"[Title/Abstract] OR "Aedes Aegypti"[Title/Abstract] OR "Aedes Albopictus"[Title/Abstract] | 81,195 |
| 2 | "Philippines"[Mesh] "Philipines"[Title/Abstract] "Phillipines"[Title/Abstract] OR "Phillippines"[Title/Abstract] OR "Republic of the Philippines"[Title/Abstract] | 106 |
| 3 | #1 AND #2 | 10 |
| **Japan (Search date: August 27, 2024)** | | |
| 1 | "Dengue"[Mesh] OR "Arbovirus Infections"[Mesh] OR "Aedes"[Mesh] OR "Severe Dengue"[Mesh] OR "Severe Dengue"[Title/Abstract] OR "Aedes"[Title/Abstract] OR "Dengue"[Title/Abstract] OR "Arbovirus Infection"[Title/Abstract] OR "Dengue Fever"[Title/Abstract] OR "Classical Dengue"[Title/Abstract] OR "Break-Bone Fever"[Title/Abstract] OR "Break Bone Fever"[Title/Abstract] OR "Breakbone Fever"[Title/Abstract] OR "Dengue Hemorrhagic Fever"[Title/Abstract] OR "Singapore Hemorrhagic Fever"[Title/Abstract] OR "Thai Hemorrhagic Fever"[Title/Abstract] OR "Philippine Hemorrhagic Fever"[Title/Abstract] OR "Dengue Shock Syndrome"[Title/Abstract] OR "Mosquito-borne Flavivirus"[Title/Abstract] OR "Dengue Outbreak"[Title/Abstract] OR "Aedes Aegypti"[Title/Abstract] OR "Aedes Albopictus"[Title/Abstract] | 81,195 |
| 2 | Japan[Mesh] OR "Japanese"[Title/Abstract] OR "Bonin Islands"[Title/Abstract] OR "Japan"[Title/Abstract] | 325,216 |
| 3 | #1 AND #2 | 5,106 |
| **Brazil (Search date: August 27, 2024)** | | |
| 1 | "Dengue"[Mesh] OR "Arbovirus Infections"[Mesh] OR "Aedes"[Mesh] OR "Severe Dengue"[Mesh] OR "Severe Dengue"[Title/Abstract] OR "Aedes"[Title/Abstract] OR "Dengue"[Title/Abstract] OR "Arbovirus Infection"[Title/Abstract] OR "Dengue Fever"[Title/Abstract] OR "Classical Dengue"[Title/Abstract] OR "Break-Bone Fever"[Title/Abstract] OR "Break Bone Fever"[Title/Abstract] OR "Breakbone Fever"[Title/Abstract] OR "Dengue Hemorrhagic Fever"[Title/Abstract] OR "Singapore Hemorrhagic Fever"[Title/Abstract] OR "Thai Hemorrhagic Fever"[Title/Abstract] OR "Philippine Hemorrhagic Fever"[Title/Abstract] OR "Dengue Shock Syndrome"[Title/Abstract] OR "Mosquito-borne Flavivirus"[Title/Abstract] OR "Dengue Outbreak"[Title/Abstract] OR "Aedes Aegypti"[Title/Abstract] OR "Aedes Albopictus"[Title/Abstract] | 81,195 |
| 2 | Brazil[Title/Abstract] OR Brazil[Mesh] OR "Brazilian"[Title/Abstract] OR "Sao Paulo"[Title/Abstract] OR "Rio de Janeiro"[Title/Abstract] | 178,771 |
| 3 | #1 AND #2 | 4,362 |
| **Paraguay (Search date: August 27, 2024)** | | |
| 1 | "Dengue"[Mesh] OR "Arbovirus Infections"[Mesh] OR "Aedes"[Mesh] OR "Severe Dengue"[Mesh] OR "Severe Dengue"[Title/Abstract] OR "Aedes"[Title/Abstract] OR "Dengue"[Title/Abstract] OR "Arbovirus Infection"[Title/Abstract] OR "Dengue Fever"[Title/Abstract] OR "Classical Dengue"[Title/Abstract] OR "Break-Bone Fever"[Title/Abstract] OR "Break Bone Fever"[Title/Abstract] OR "Breakbone Fever"[Title/Abstract] OR "Dengue Hemorrhagic Fever"[Title/Abstract] OR "Singapore Hemorrhagic Fever"[Title/Abstract] OR "Thai Hemorrhagic Fever"[Title/Abstract] OR "Philippine Hemorrhagic Fever"[Title/Abstract] OR "Dengue Shock Syndrome"[Title/Abstract] OR "Mosquito-borne Flavivirus"[Title/Abstract] OR "Dengue Outbreak"[Title/Abstract] OR "Aedes Aegypti"[Title/Abstract] OR "Aedes Albopictus"[Title/Abstract] | 81,195 |
| 2 | Paraguay[Title/Abstract] OR Paraguay[Mesh] OR "Republic of Paraguay"[Title/Abstract] | 2,242 |
| 3 | #1 AND #2 | 75 |
| **France (Search date: August 28, 2024)** | | |
| 1 | "Dengue"[Mesh] OR "Arbovirus Infections"[Mesh] OR "Aedes"[Mesh] OR "Severe Dengue"[Mesh] OR "Severe Dengue"[Title/Abstract] OR "Aedes"[Title/Abstract] OR "Dengue"[Title/Abstract] OR "Arbovirus Infection"[Title/Abstract] OR "Dengue Fever"[Title/Abstract] OR "Classical Dengue"[Title/Abstract] OR "Break-Bone Fever"[Title/Abstract] OR "Break Bone Fever"[Title/Abstract] OR "Breakbone Fever"[Title/Abstract] OR "Dengue Hemorrhagic Fever"[Title/Abstract] OR "Singapore Hemorrhagic Fever"[Title/Abstract] OR "Thai Hemorrhagic Fever"[Title/Abstract] OR "Philippine Hemorrhagic Fever"[Title/Abstract] OR "Dengue Shock Syndrome"[Title/Abstract] OR "Mosquito-borne Flavivirus"[Title/Abstract] OR "Dengue Outbreak"[Title/Abstract] OR "Aedes Aegypti"[Title/Abstract] OR "Aedes Albopictus"[Title/Abstract] | 81,204 |
| 2 | France[Title/Abstract] OR France[Mesh] OR "French Republic"[Title/Abstract] OR "St. Pierre and Miquelon"[Title/Abstract] OR "Miquelon and Saint Pierre"[Title/Abstract] OR "Miquelon and St. Pierre"[Title/Abstract] OR "Saint Pierre and Miquelon"[Title/Abstract] OR "Corsica"[Title/Abstract] | 151,946 |
| 3 | #1 AND #2 | 738 |
| **Portugal (Search date: August 28, 2024)** | | |
| 1 | "Dengue"[Mesh] OR "Arbovirus Infections"[Mesh] OR "Aedes"[Mesh] OR "Severe Dengue"[Mesh] OR "Severe Dengue"[Title/Abstract] OR "Aedes"[Title/Abstract] OR "Dengue"[Title/Abstract] OR "Arbovirus Infection"[Title/Abstract] OR "Dengue Fever"[Title/Abstract] OR "Classical Dengue"[Title/Abstract] OR "Break-Bone Fever"[Title/Abstract] OR "Break Bone Fever"[Title/Abstract] OR "Breakbone Fever"[Title/Abstract] OR "Dengue Hemorrhagic Fever"[Title/Abstract] OR "Singapore Hemorrhagic Fever"[Title/Abstract] OR "Thai Hemorrhagic Fever"[Title/Abstract] OR "Philippine Hemorrhagic Fever"[Title/Abstract] OR "Dengue Shock Syndrome"[Title/Abstract] OR "Mosquito-borne Flavivirus"[Title/Abstract] OR "Dengue Outbreak"[Title/Abstract] OR "Aedes Aegypti"[Title/Abstract] OR "Aedes Albopictus"[Title/Abstract] | 81,204 |
| 2 | Portugal[Title/Abstract] OR Portugal[Mesh] OR "Portuguese Republic"[Title/Abstract] OR "Madeira Island"[Title/Abstract] | 24,947 |
| 3 | #1 AND #2 | 157 |
| **Spain (Search date: August 28, 2024)** | | |
| 1 | "Dengue"[Mesh] OR "Arbovirus Infections"[Mesh] OR "Aedes"[Mesh] OR "Severe Dengue"[Mesh] OR "Severe Dengue"[Title/Abstract] OR "Aedes"[Title/Abstract] OR "Dengue"[Title/Abstract] OR "Arbovirus Infection"[Title/Abstract] OR "Dengue Fever"[Title/Abstract] OR "Classical Dengue"[Title/Abstract] OR "Break-Bone Fever"[Title/Abstract] OR "Break Bone Fever"[Title/Abstract] OR "Breakbone Fever"[Title/Abstract] OR "Dengue Hemorrhagic Fever"[Title/Abstract] OR "Singapore Hemorrhagic Fever"[Title/Abstract] OR "Thai Hemorrhagic Fever"[Title/Abstract] OR "Philippine Hemorrhagic Fever"[Title/Abstract] OR "Dengue Shock Syndrome"[Title/Abstract] OR "Mosquito-borne Flavivirus"[Title/Abstract] OR "Dengue Outbreak"[Title/Abstract] OR "Aedes Aegypti"[Title/Abstract] OR "Aedes Albopictus"[Title/Abstract] | 81,204 |
| 2 | Spain[Title/Abstract] OR Spain[Mesh] OR "Spanish Population"[Title/Abstract] OR "Balearic Islands"[Title/Abstract] OR "Canary Islands"[Title/Abstract] | 125,764 |
| 3 | #1 AND #2 | 524 |
| **Peru (Search date: August 28, 2024)** | | |
| 1 | "Dengue"[Mesh] OR "Arbovirus Infections"[Mesh] OR "Aedes"[Mesh] OR "Severe Dengue"[Mesh] OR "Severe Dengue"[Title/Abstract] OR "Aedes"[Title/Abstract] OR "Dengue"[Title/Abstract] OR "Arbovirus Infection"[Title/Abstract] OR "Dengue Fever"[Title/Abstract] OR "Classical Dengue"[Title/Abstract] OR "Break-Bone Fever"[Title/Abstract] OR "Break Bone Fever"[Title/Abstract] OR "Breakbone Fever"[Title/Abstract] OR "Dengue Hemorrhagic Fever"[Title/Abstract] OR "Singapore Hemorrhagic Fever"[Title/Abstract] OR "Thai Hemorrhagic Fever"[Title/Abstract] OR "Philippine Hemorrhagic Fever"[Title/Abstract] OR "Dengue Shock Syndrome"[Title/Abstract] OR "Mosquito-borne Flavivirus"[Title/Abstract] OR "Dengue Outbreak"[Title/Abstract] OR "Aedes Aegypti"[Title/Abstract] OR "Aedes Albopictus"[Title/Abstract] | 81,204 |
| 2 | Peru[Title/Abstract] OR Peru[Mesh] OR "Peruvian"[Title/Abstract] OR "Lima"[Title/Abstract] OR "Arequipa"[Title/Abstract] OR "Cusco"[Title/Abstract] | 22,475 |
| 3 | #1 AND #2 | 380 |
| **United States (Search date: August 28, 2024)** | | |
| 1 | "Dengue"[Mesh] OR "Arbovirus Infections"[Mesh] OR "Aedes"[Mesh] OR "Severe Dengue"[Mesh] OR "Severe Dengue"[Title/Abstract] OR "Aedes"[Title/Abstract] OR "Dengue"[Title/Abstract] OR "Arbovirus Infection"[Title/Abstract] OR "Dengue Fever"[Title/Abstract] OR "Classical Dengue"[Title/Abstract] OR "Break-Bone Fever"[Title/Abstract] OR "Break Bone Fever"[Title/Abstract] OR "Breakbone Fever"[Title/Abstract] OR "Dengue Hemorrhagic Fever"[Title/Abstract] OR "Singapore Hemorrhagic Fever"[Title/Abstract] OR "Thai Hemorrhagic Fever"[Title/Abstract] OR "Philippine Hemorrhagic Fever"[Title/Abstract] OR "Dengue Shock Syndrome"[Title/Abstract] OR "Mosquito-borne Flavivirus"[Title/Abstract] OR "Dengue Outbreak"[Title/Abstract] OR "Aedes Aegypti"[Title/Abstract] OR "Aedes Albopictus"[Title/Abstract] | 81,204 |
| 2 | "United States"[Title/Abstract] OR "United States of America"[Title/Abstract] OR "America"[Title/Abstract] OR "United States"[Mesh] | 1,742,699 |
| 3 | #1 AND #2 | 7,500 |
| **Colombia (Search date: August 28, 2024)** | | |
| 1 | "Dengue"[Mesh] OR "Arbovirus Infections"[Mesh] OR "Aedes"[Mesh] OR "Severe Dengue"[Mesh] OR "Severe Dengue"[Title/Abstract] OR "Aedes"[Title/Abstract] OR "Dengue"[Title/Abstract] OR "Arbovirus Infection"[Title/Abstract] OR "Dengue Fever"[Title/Abstract] OR "Classical Dengue"[Title/Abstract] OR "Break-Bone Fever"[Title/Abstract] OR "Break Bone Fever"[Title/Abstract] OR "Breakbone Fever"[Title/Abstract] OR "Dengue Hemorrhagic Fever"[Title/Abstract] OR "Singapore Hemorrhagic Fever"[Title/Abstract] OR "Thai Hemorrhagic Fever"[Title/Abstract] OR "Philippine Hemorrhagic Fever"[Title/Abstract] OR "Dengue Shock Syndrome"[Title/Abstract] OR "Mosquito-borne Flavivirus"[Title/Abstract] OR "Dengue Outbreak"[Title/Abstract] OR "Aedes Aegypti"[Title/Abstract] OR "Aedes Albopictus"[Title/Abstract] | 81,204 |
| 2 | Colombia[Title/Abstract] OR Colombia[Mesh] OR "Republic of Colombia"[Title/Abstract] | 22,013 |
| 3 | #1 AND #2 | 848 |
| **Australia (Search date: August 28, 2024)** | | |
| 1 | "Dengue"[Mesh] OR "Arbovirus Infections"[Mesh] OR "Aedes"[Mesh] OR "Severe Dengue"[Mesh] OR "Severe Dengue"[Title/Abstract] OR "Aedes"[Title/Abstract] OR "Dengue"[Title/Abstract] OR "Arbovirus Infection"[Title/Abstract] OR "Dengue Fever"[Title/Abstract] OR "Classical Dengue"[Title/Abstract] OR "Break-Bone Fever"[Title/Abstract] OR "Break Bone Fever"[Title/Abstract] OR "Breakbone Fever"[Title/Abstract] OR "Dengue Hemorrhagic Fever"[Title/Abstract] OR "Singapore Hemorrhagic Fever"[Title/Abstract] OR "Thai Hemorrhagic Fever"[Title/Abstract] OR "Philippine Hemorrhagic Fever"[Title/Abstract] OR "Dengue Shock Syndrome"[Title/Abstract] OR "Mosquito-borne Flavivirus"[Title/Abstract] OR "Dengue Outbreak"[Title/Abstract] OR "Aedes Aegypti"[Title/Abstract] OR "Aedes Albopictus"[Title/Abstract] | 81,204 |
| 2 | Australia[Title/Abstract] OR Australia[Mesh] OR "Commonwealth of Australia"[Title/Abstract] OR "Canton and Enderbury Islands"[Title/Abstract] OR "Christmas Island"[Title/Abstract] | 227,745 |
| 3 | #1 AND #2 | 1,417 |
| **Iran (Search date: October 20, 2024)** | | |
| 1 | "Dengue"[Mesh] OR "Arbovirus Infections"[Mesh] OR "Aedes"[Mesh] OR "Severe Dengue"[Mesh] OR "Severe Dengue"[Title/Abstract] OR "Aedes"[Title/Abstract] OR "Dengue"[Title/Abstract] OR "Arbovirus Infection"[Title/Abstract] OR "Dengue Fever"[Title/Abstract] OR "Classical Dengue"[Title/Abstract] OR "Break-Bone Fever"[Title/Abstract] OR "Break Bone Fever"[Title/Abstract] OR "Breakbone Fever"[Title/Abstract] OR "Dengue Hemorrhagic Fever"[Title/Abstract] OR "Singapore Hemorrhagic Fever"[Title/Abstract] OR "Thai Hemorrhagic Fever"[Title/Abstract] OR "Philippine Hemorrhagic Fever"[Title/Abstract] OR "Dengue Shock Syndrome"[Title/Abstract] OR "Mosquito-borne Flavivirus"[Title/Abstract] OR "Dengue Outbreak"[Title/Abstract] OR "Aedes Aegypti"[Title/Abstract] OR "Aedes Albopictus"[Title/Abstract] | 81,808 |
| 2 | Iran[Title/Abstract] OR Iran[Mesh] OR "Islamic Republic of Iran"[Title/Abstract] OR "Tehran"[Title/Abstract] OR "Isfahan"[Title/Abstract] OR "Khorasan"[Title/Abstract] OR "Fars"[Title/Abstract] OR "Mazandaran"[Title/Abstract] OR "East Azerbaijan"[Title/Abstract] OR "West Azerbaijan"[Title/Abstract] OR "Khuzestan"[Title/Abstract] OR "Golestan"[Title/Abstract] | 76755 |
| 3 | #1 AND #2 | 200 |
